# A case of ichthyosiform mycosis fungoides

**DOI:** 10.1016/j.jdcr.2026.08.012

**Published:** 2026-08-17

**Authors:** Hee Jeong Han, Jieun Maeng, You Chan Kim

**Affiliations:** Department of Dermatology, Ajou University School of Medicine, Suwon, Republic of Korea

**Keywords:** acquired ichthyosis, ichthyosiform mycosis fungoides, mycosis fungoides

## Case description

A 64-year-old man presented with a 20-year history of generalized ichthyosiform patches on the trunk and extremities. He had been previously diagnosed with ichthyosis vulgaris at another hospital and had received intermittent treatment. His face, palms, soles, and flexural areas were spared, and there were no classic patches, plaques, or tumors (Fig 1). He denied pruritus or pain. Physical examination revealed no palpable peripheral lymphadenopathy or organomegaly. Laboratory evaluations, including complete blood count, differential leukocyte count, erythrocyte sedimentation rate, and serum chemistry, were within normal limits. Formal Modified Severity-Weighted Assessment Tool scoring, tumor, node, metastasis blood staging, and computed tomography or positron emission tomography–computed tomography were not performed. Skin biopsies from the abdomen and legs revealed focally compact orthokeratosis, a reduced epidermal granular layer, and lichenoid lymphocytic infiltration with atypical lymphocyte epidermotropism (Fig 2). Immunohistochemical staining showed a T-cell phenotype (CD2^+^, CD3^+^, CD4^+^, focally CD30^+^, CD8^−^, and CD20^−^) in epidermal and dermal lymphocytes (Fig 3). The patient was treated with narrowband ultraviolet B phototherapy twice weekly plus topical corticosteroids. He continued phototherapy elsewhere near his residence for 2 years, so the exact dose and cumulative dose were unavailable. At follow-up at our clinic, he showed a partial clinical response on physical examination, with decreased lesion extent and improved scaling, ichthyosiform change, erythema, and hyperpigmentation.

**Fig 1:**
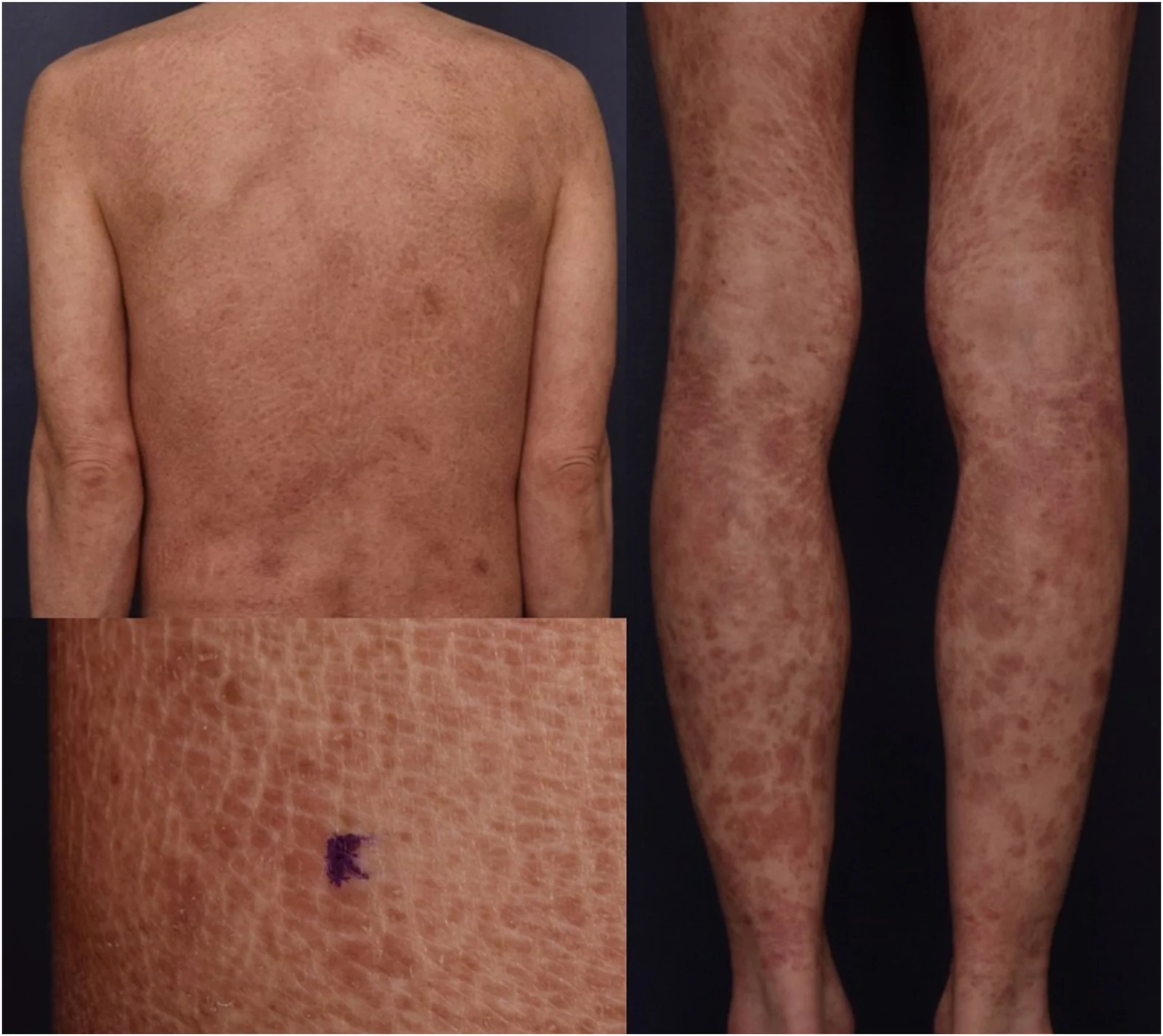
Generalized ichthyosiform eruptions on the trunk and extremities.

**Fig 2:**
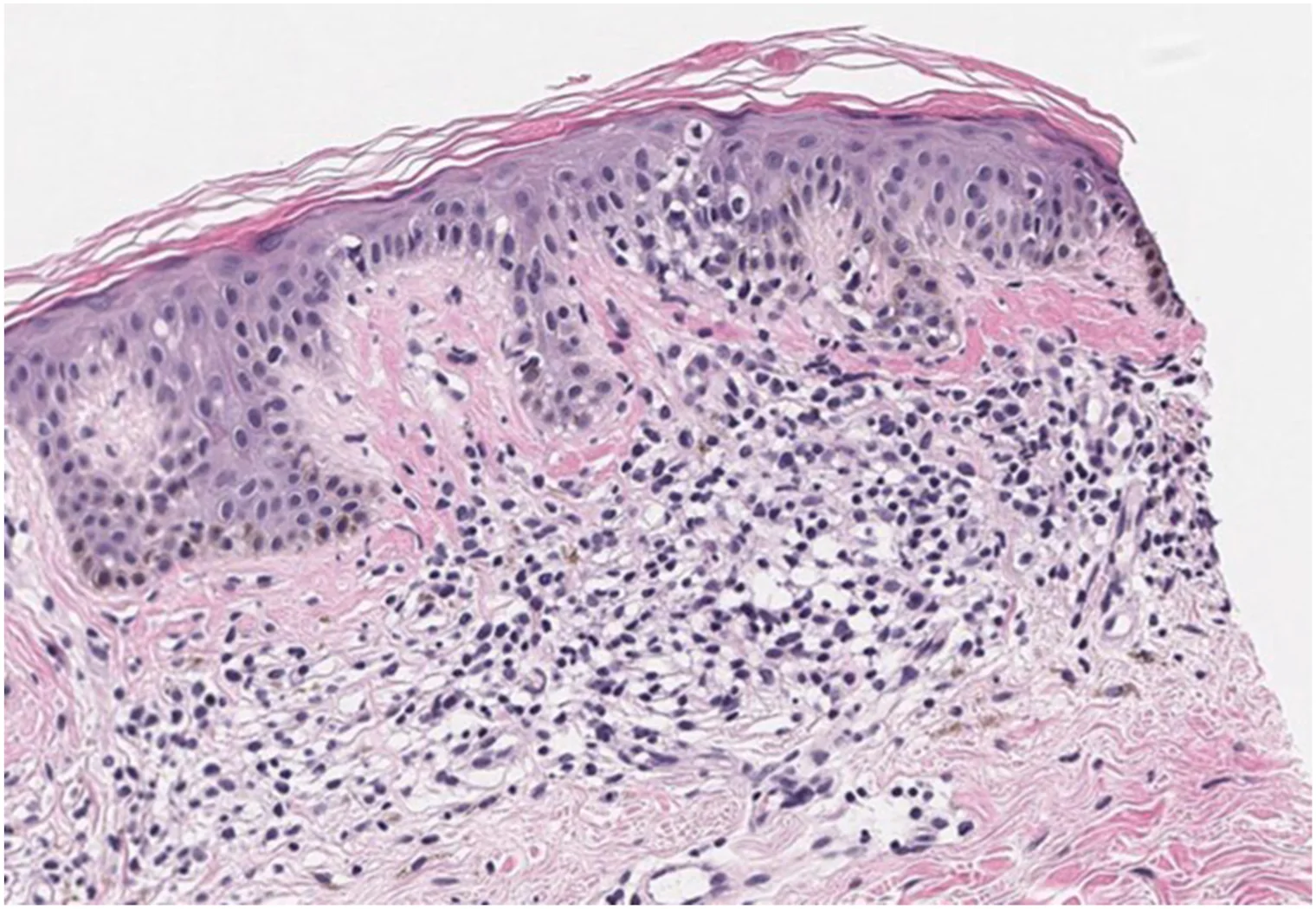
Histopathology revealed focally compact orthokeratosis, a reduction in the granular layer of the epidermis, and lichenoid lymphocytic infiltration with epidermotropism of atypical lymphocytes. (Original magnification ×200).

**Fig 3:**
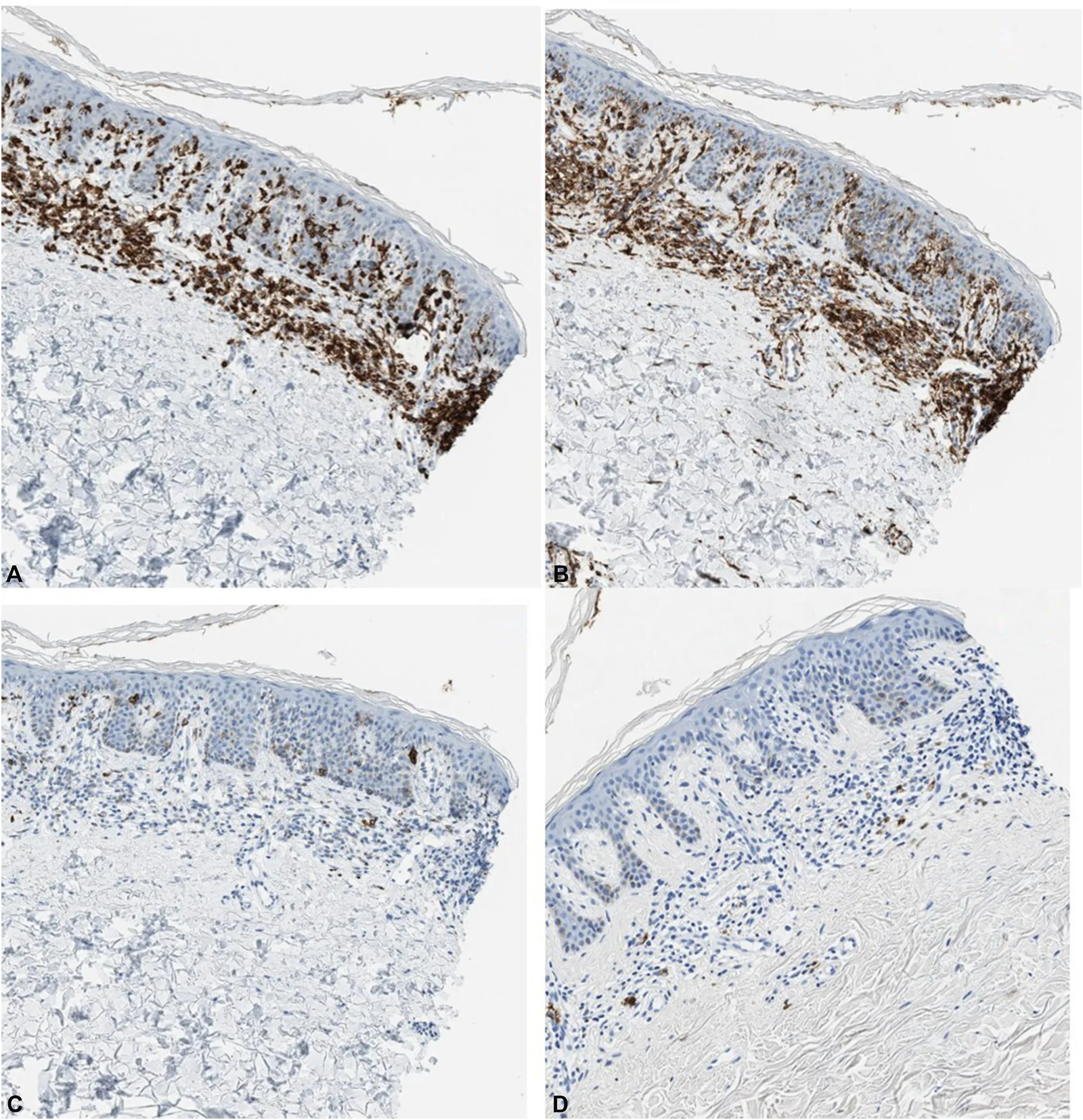
Immunohistochemical staining indicated a T-cell phenotype: **(A)** CD3^+^, **(B)** CD4^+^, **(C)** focally CD30^+^, and **(D)** CD20^−^. (Original magnification ×200).

### Question: What is the most likely diagnosis?

A. Ichthyosis vulgaris
B. Acquired ichthyosis secondary to mycosis fungoides
C. Ichthyosiform mycosis fungoides
D. Pityriasis rubra pilaris
E. Sézary syndrome

**Answer discussion:** The correct answer is C, ichthyosiform mycosis fungoides (IMF). The World Health Organization–European Organization for Research and Treatment of Cancer classification recognizes 3 variants of mycosis fungoides (MF), but additional atypical forms have been described, and Hodak and Amitay-Laish^1^ categorized MF into 15 subtypes. IMF, first described in 1996, can present as localized or generalized ichthyosiform eruptions, with or without typical MF lesions.^2^^,^^3^ Histologically, IMF overlaps with ichthyosis and MF, showing compact orthokeratosis, hypogranulosis, and atypical lymphocyte epidermotropism.^3^ Most cases show a T-helper immunophenotype, typically with CD3 and CD4 positivity, often with loss of CD7.^3^ In this patient, the ichthyosiform lesions showed epidermotropism of atypical T lymphocytes with CD3 and CD4 positivity, supporting IMF. The main diagnostic pitfall is acquired ichthyosis secondary to MF. Acquired ichthyosis may occur with malignancies, including lymphoma, MF, endocrine or metabolic disorders, and drug reactions.^2^^,^^3^ However, this patient had only generalized ichthyosiform lesions classic patches, plaques, or tumors, favoring IMF. IMF accounts for ∼4% of MF cases, but an isolated ichthyosiform presentation is rare.^4^ Delineating IMF from conventional MF is clinically relevant because its morphology may mimic benign ichthyoses or eczematous dermatoses, delaying biopsy and diagnosis. Recognition of IMF does not mandate distinct treatment because management and prognosis depend primarily on stage and treatment response. Early or skin-limited disease can be treated with skin-directed therapies, including topical corticosteroids and phototherapy. Methotrexate, oral retinoids, and retinoid-phototherapy combinations have also been used.^5^ Although IMF generally has a favorable prognosis, the independent prognostic significance of ichthyosiform morphology remains unclear. This case emphasizes that persistent or atypical ichthyosiform eruptions warrant early biopsy, clinicopathologic correlation, and follow-up to prevent diagnostic delay.

## Conflict of interest

None disclosed.
